# Predictors of Malignancy in Patients With Indeterminate Biliary Strictures and Atypical Biliary Cytology: Results From Retrospective Cohort Study

**DOI:** 10.1093/jcag/gwaa043

**Published:** 2021-03-18

**Authors:** Ali Alali, Maria Moris, Myriam Martel, Catherine Streutker, Maria Cirocco, Jeffrey Mosko, Paul Kortan, Alan Barkun, Gary R May

**Affiliations:** 1 The Center for Therapeutic Endoscopy and Endoscopic Oncology, St. Michael’s Hospital, University of Toronto, Toronto, Ontario, Canada; 2 Haya Al-Habeeb Gastroenterology and Hepatology Center, Mubarak Al-Kabeer Hospital, Jabriya, Kuwait; 3 Digestive Disease Department, Marqués de Valdecilla University Hospital, Cantabria University, Santander, Spain; 4 Division of Gastroenterology, McGill University Health Center, McGill University, Montreal, Quebec, Canada; 5 Department of Laboratory Medicine, St. Michael’s Hospital, University of Toronto, Toronto, Ontario, Canada

**Keywords:** Brush, Cholangiocarcinoma, Cytology, ERCP, Malignancy, Pancreatic cancer

## Abstract

**Background:**

Atypical cellular features are commonly encountered in patients with indeterminate biliary strictures, which are nondiagnostic of malignancy yet cannot rule it out. This study aims to identify clinical features that could discriminate patients with indeterminate biliary strictures and atypical biliary cytology who may harbor underlying malignancy.

**Methods:**

All patients with an indeterminate biliary stricture and an atypical brush cytology obtained during endoscopic brushings were identified in a large tertiary-care center. Demographical information, clinical data and the final pathological diagnosis were collected. The study cohort was divided based on the final diagnosis into benign and malignant groups. Descriptive and multivariable analyses were performed.

**Results:**

A total of 151 patients were included in the analysis. Of these, 62.9% were males with mean age of 61.7 ± 16.4 years. Overall, there was an almost equal distribution of patients in the benign and malignant groups. Older age (≥65 years), jaundice, weight loss, intrahepatic biliary and pancreatic duct dilation, double-duct sign and presence of a mass were associated with malignancy in the univariate analysis. However, only older age (odds ratio [OR] 1.02, 95% confidence interval [CI] 1.00 to 1.03), jaundice (OR 3.33, 95% CI 1.11 to 9.98) and presence of a mass (OR 12.10, 95% CI 4.94 to 29.67) were significantly associated with malignancy in the multivariate analysis. High CA19-9 was associated with malignancy only in patients with primary sclerosing cholangitis.

**Conclusion:**

In patients with indeterminate biliary stricture and atypical brush cytology, older age, jaundice and presence of a mass are significant predictors of malignancy. Patients with such characteristics need prompt evaluation to rule out underlying malignancy.

## Introduction

Endoscopic retrograde cholangiopancreatography (ERCP) is a common endoscopic procedure that is used to diagnose and treat patients with biliary strictures. Biliary strictures can be secondary to benign or malignant etiologies and pathological diagnosis is crucial to facilitate further management (1). Approximately 15 to 24% of patients undergoing surgical resection for suspected biliary malignancy have benign etiology (2–6), exposing these patients to unnecessary risks. Therefore, preoperative diagnosis of malignancy in a biliary stricture is essential before undergoing any surgical procedure.

Typically, patients with biliary strictures undergo ERCP to obtain tissue through cytology brushings or biopsy forceps (7). Even though these modalities have excellent specificity (99%), they suffer from poor sensitivity (45% and 48% for brush cytology and biopsy forceps, respectively) (8). One of the main challenges encountered when dealing with biliary strictures is ‘indeterminate strictures’. Traditionally, biliary strictures have been considered to be indeterminate when a diagnosis cannot be made after basic laboratory work-up, imaging studies and ERCP with biliary tissue sampling, usually with a cytology brush. Not uncommonly, tissue obtained via cytology brushings may show scarce abnormal cells insufficient to be labelled as malignant and therefore classified as atypical. Alternatively, there may be more widespread changes in a background of inflammation or history of inflammation (primary sclerosing cholangitis [PSC], stones) where inflammatory changes are indistinguishable from neoplasia. The frequency of atypical biliary brushings ranges between 8.1 to 25.8% in some series (9–12). These ‘atypical cytology’ results represent a clinical dilemma in patients with indeterminate biliary strictures as some may harbor malignancy and warrant surgical resection or chemotherapy, whereas others may simply represent inflammatory/benign etiology. In particular, patients with chronic inflammatory biliary conditions such as PSC are even more challenging given the lower brush cytology sensitivity due to the marked inflammatory and reactive changes (13).

Given the high frequency of atypical cytology results obtained from ERCP, it is crucial to identify other factors to help establishing a more precise diagnosis and, therefore, avoid unnecessary surgical interventions that can be harmful to the patient and costly to the health system. The aim of this study is to identify such factors that may help to differentiate benign from malignant etiologies in patients with atypical biliary cytology.

## METHODS

This study was approved by the St. Michael’s Hospital Institutional Review Board.

### Study Design

A retrospective chart review was conducted, where all patients with a biliary stricture and an ‘atypical’ brush cytology obtained during ERCP from January 2011 to June 2016 at St. Michael’s hospital in Toronto, a tertiary hospital, were identified.

An initial search in the cytopathology laboratory information system was conducted for all bile duct brushing samples obtained during the study period. Then, all ‘atypical’ cytology results were identified. Relevant clinical patient information was collected from the hospital electronic computer system including: patient demographics, clinical data (including symptoms, laboratory results and follow-up data), imaging findings (including imaging modality, presence of a mass lesion or ductal dilation) and final pathological diagnosis. The final pathological diagnosis was obtained by tissue acquisition either through surgical/repeat brushing cytology specimen or using another means of obtaining biopsy (e.g., percutaneous biopsy). The final study cohort was divided into benign and malignant groups based on their final diagnosis.

Patients with a biliary stricture who underwent ERCP and had ‘atypical’ biliary cytology brushing results were included in the study if they were at least 18 years of age and had at least 6 months of follow-up after the date of the ERCP. All patients with insufficient documentation were excluded from the study.

### Definitions

The ‘benign group’ is defined as any patient who had a benign pathology identified in any tissue specimen obtained during follow-up visits including repeat brushing, tissue obtained by percutaneous biopsy, Endoscopic Ultrasound guided Fine Needle Aspiration/Biopsy (EUS-FNA or EUS-FNB) or surgical specimen. It also included patients who were followed-up for at least 6 months and did not manifest any evidence of malignancy during this period. The ‘malignant group’ was defined as any patient who had a malignant tissue obtained during follow-up (same modalities as above). Also, the malignant group included patients who had imaging evidence of metastatic disease where tissue was not required for further management of the patient.

### Patients

The study included all adult patients who were referred for ERCP for the management of a biliary stricture. Patients underwent ERCP in a standard fashion and any stricture identified was brushed using a cytology brush. All patients underwent pre-ERCP imaging including at least one of transabdominal ultrasound, computed tomography (CT), abdominal magnetic resonance imaging (MRI), and/or endoscopic ultrasound (EUS). All ERCP images were retrieved and reviewed again to confirm the location and the length of the stricture to ensure standardization and accuracy of the data.

### Cytology Results

All brush cytology specimens were reviewed by experienced pathologists. The pathologist was aware of the indication of the procedure and the site of the biliary stricture. All pathologists had access to the patient’s medical record for review if needed. The Standardized Terminology and Nomenclature for Pancreaticobiliary Cytology (STNPC) system was used to classify the cytology specimen. Atypical cytology was defined as ‘cells with cytoplasmic, nuclear, or architectural features not consistent with normal or reactive cellular components of the pancreas or bile ducts and insufficient features to classify them as neoplasm or suspicious for a high grade malignancy’ (14). Most specimens were reviewed by at least another pathologist to confirm the diagnosis of ‘atypical’ cytology. Only brush cytology specimens that were labelled as ‘atypical’ were included in the analysis. Specimens that had a diagnosis of ‘benign’, ‘malignant’ or ‘suspicious’ were not included.

### Outcomes

The main outcome measure was to identify variables associated with malignancy in patients with biliary stricture and an atypical cytology results. These variables include age, gender, symptoms, bilirubin level, CA19-9 level, biliary and/or pancreatic duct dilation and the presence of a mass. In addition, sub-group analysis of patient with PSC was planned a priori.

### Statistical Analysis

Descriptive statistics were carried out and reported as mean ± standard deviation or percentages. Between-group comparisons were carried out between malignant and benign diagnosis for all variables using the chi-square test and *t*-test, where appropriate. Multivariable analyses were performed to identify variables associated with malignancy. A statistical significance threshold of *P* = 0.05 was adopted. All analyses were performed using SAS software version 9.4 (SAS Institute, Cary, NC, USA).

## RESULTS

**Figure 1.**
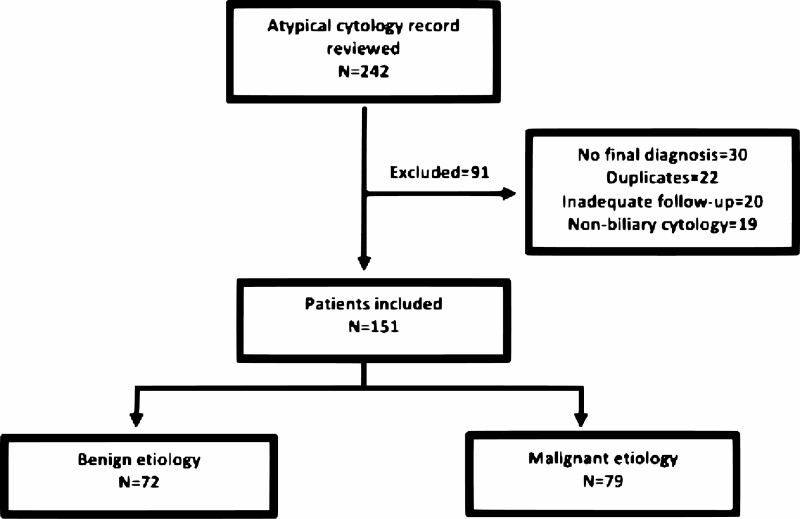
Flow chart of patients included in the study.

Initially, 242 atypical cytology brush results were identified from January 2011 to June 2016. After excluding duplicates, nonbiliary cytology and patients with no final diagnosis or inadequate follow-up time, a total of 151 patients were included (Figure 1).

### Demographics

The mean age was 61.7 ± 16.4 years with 37.1% females. The majority of patients were symptomatic 96.7%. The most common symptoms were jaundice (108/151, 71.5%) and abdominal pain (88/151, 58.3%). All patients had at least one imaging modality with CT being the most commonly used (127/151, 84.1%). The most common location of the stricture was distal common bile duct (CBD) (107/151, 70.9%) and the mean stricture length was 19.4 ± 13.7 mm. See Table 1 for detailed baseline characteristics.

**Table 1.** Baseline characteristics (*n* = 151 patients)

	Value
Age, years (mean ± SD)	61.7 ± 16.4
Gender, *n* (%)	
Male	95 (62.9)
Female	56 (37.1)
Smoking, *n* (%)	
Never	92 (68.7)
Past	24 (17.9)
Current	18 (13.4)
Symptoms, *n* (%)	
Yes	146 (96.7)
No	5 (3.3)
Abdominal pain, *n* (%)	88 (58.3)
Jaundice, *n* (%)	108 (71.5)
Pruritus, *n* (%)	38 (25.2)
Weight loss, *n* (%)	45 (29.8)
Fever, *n* (%)	23 (15.2)
Diarrhea, *n* (%)	3 (2.0)
Pre-ERCP imaging, *n* (%)	
Ultrasound	126 (83.4)
CT	127 (84.1)
MRI	109 (72.2)
EUS	48 (31.8)
Pathology, *n* (%)	
Benign	72 (47.7)
Malignant	79 (52.3)
Stricture length, mm (mean ± SD)	19.4 ± 13.7
Stricture characteristics, *n* (%)	
Distal CBD	107 (70.9)
Mid-CBD	14 (9.3)
Proximal CBD	7 (4.6)
Hilar	20 (13.3)
Intrahepatic ducts	3 (2.0)

CBD, common bile duct; ERCP, Endoscopic retrograde cholangiopancreatography; EUS, Endoscopic ultrasound; MRI, Magnetic resonance imaging.

### Final Outcome

Final diagnosis was established by a number of different approaches as described in Table 2. In this cohort, a higher proportion of patients were diagnosed with a malignant stricture (79/151, 52.3%) than benign stricture (72/151, 47.7%). The most common malignant etiology was primary pancreaticobiliary malignancy including pancreatic adenocarcinoma (49/79, 62.0%), cholangiocarcinoma (20/79, 25.3%) and ampullary cancer (6/79, 7.6%) followed by metastatic cancer (4/79, 5.1%).

**Table 2.** Mode of diagnosis and final pathological diagnosis (*n* = 151 patients)

Mode of diagnosis	*N* (%)
Follow-up	57 (37.8)
Surgery	39 (25.8)
Biopsy	29 (19.2)
Endoscopic ultrasound-Fine needle aspiration	14 (9.3)
Advanced cancer	10 (6.6)
Repeated brushing	2 (1.3)
Pathology	*N* (%)
Ampullary cancer	6 (4.0)
Autoimmune pancreatitis	9 (6.0)
Cholangiocarcinoma	20 (13.3)
Chronic pancreatitis	10 (6.6)
Metastatic cancer	4 (2.7)
Other benign	32 (21.2)
Pancreatic adenocarcinoma	49 (32.5)
Postoperative stricture	1 (0.7)
Primary sclerosing cholangitis	20 (13.3)

Overall, 72/151 patients (47.7%) had a benign biliary stricture. The most common benign etiology was PSC (20/72,27.8%) followed by chronic pancreatitis (10/72, 13.9%). The mean clinical follow-up time for the patients with no tissue diagnosis was 931 days (range 182 to 1940 days).

### Variables Associated With Malignancy

Several factors were identified that significantly increased the probability of malignancy. These variables include increasing age, weight loss, absence of abdominal pain, higher levels of bilirubin, the presence of mass on imaging, double-duct sign, and intrahepatic biliary and/or pancreatic duct dilation. However, after adjusting for multiple potential confounders, multivariable analysis showed that only age ≥ 65 years (odds ratio [OR] 1.02, 95% confidence interval [CI] 1.00 to 1.03), jaundice (OR 3.33, 95% CI 1.11 to 9.98) and the presence of a mass on imaging (OR 12.10, 95% CI 4.94 to 29.67) were found to be significantly associated with underlying malignancy. See Tables 3 and 4 for detailed results.

**Table 3.** Univariable analysis (overall cohort)

Variable	Benign *N* = 72	Malignant *N* = 79	*P*-value
Age, years (mean ± SD)	55.7 ± 18.7	67.1 ± 12.4	<0.01
Gender			
Male, *n* (%)	50 (69.4)	45 (57.0)	0.10
Female, *n* (%)	22 (30.6)	34 (43.0)	
Abdominal pain, *n* (%)	53 (73.6)	35 (44.3)	<0.01
Jaundice, *n* (%)	41 (56.9)	66 (83.5)	<0.01
Weight loss, *n* (%)	13 (18.1)	32 (40.5)	<0.01
Smoking, *n* (%)			
Never	41 (67.2)	51 (70.8)	0.70
Current or past	20 (32.8)	21 (29.2)	0.70
Laboratory			
Alkaline phosphatase (IU/L), mean ± SD	396.2 ± 386.0	460.3 ± 322.2	0.3
CA19-9 (U/mL), mean ± SD	152.4 ± 763.3	1253.4 ± 5285.8	0.1
Bilirubin (μmol/L), mean ± SD	81.4 ± 119.9	152.5 ± 135.9	<0.01
Imaging			
Presence of a mass, *n* (%)	19 (26.4)	67 (84.8)	<0.01
Extrahepatic biliary dilation, *n* (%)	50 (69.4)	64 (81.0)	0.1
Pancreatic duct dilation, *n* (%)	16 (22.2)	36 (45.6)	<0.01
Intrahepatic biliary dilation, *n* (%)	58 (80.6)	76 (96.2)	<0.01
Double duct sign, *n* (%)	16 (22.2)	34 (43.0)	<0.01

**Table 4.** Multivariable analysis for variables associated with malignancy

Odds ratio estimates			
Variable	Point estimate	95% confidence limits	
Age ≥65 years	1.02	1.00	1.03
Gender (male)	2.33	0.93	5.83
Abdominal pain	0.46	0.18	1.18
Jaundice	3.33	1.11	9.98
Pancreatic duct dilation	2.38	0.91	6.24
Presence of a mass	12.10	4.94	29.67

### Primary Sclerosing Cholangitis

The characteristics of patients with underlying PSC are shown in Table 5. Overall, 23 patients (15.2%) in the cohort had underlying diagnosis of PSC. The mean follow-up time was 967 days (range 185 to 1701). Three of the 23 (13.0%) had a diagnosis of malignancy (2 cholangiocarcinoma and 1 ampullary cancer). The mean age of PSC patients with malignant outcome was 62.6 years compared to a mean age of 39.6 years in patients with PSC and benign outcome (*P* < 0.001). A mass was identified in all patients with malignancy (3/3, 100%) compared to only one patient in the benign group (1/20, 5.0%). In addition, patients with malignant outcome had significantly higher CA19-9 value compared to patients with no underlying malignancy. Malignancy was diagnosed in all three patients within a year from the index ERCP (mean 323 days, range 211 to 500).

**Table 5.** Univariable analysis of predictors of malignancy (PSC cohort)

Variable	Benign group (*n* = 20)	Malignant group (*n* = 3)	*P*-value
Demographics			
Age, years (mean ± SD)	39.6 ± 14.7	62.6 ± 3.3	<0.001
Female, *n* (%)	8 (40.0)	1 (33.3)	0.8
Abdominal pain, *n* (%)	10 (50.0)	2 (66.6)	0.7
Jaundice, *n* (%)	9 (45.0)	3 (100.0)	0.07
Weight loss, *n* (%)	2 (10.0)	1 (33.3)	0.3
Laboratory			
Alkaline phosphatase (IU/L), mean ± SD	339.3 ± 247.6	393 ± 231.4	0.7
CA19-9 (U/mL), mean ± SD	22.5 ± 18.6	2179 ± 412.2	<0.001
Bilirubin (μmol/L), mean ± SD	97.5 ± 140.9	160 ± 152.2	0.5
Imaging			
Presence of a mass, *n* (%)	1 (5.0)	3 (100.0)	<0.001
Intrahepatic biliary dilation, *n* (%)	15 (75.0)	2 (66.6)	0.76

### Tumor Marker CA19-9

CA19-9 value was available in 102 patients (67.5%). The mean CA19-9 serum value in the benign group was 152.4 IU/mL which was numerically but not statistically significantly lower than the malignancy group (1253.4 IU/mL, *P* = 0.1). However, as mentioned above, the CA19-9 was significantly higher in the sub-group of patients with PSC and underlying malignancy compared to the benign one.

## Discussion

Atypical biliary cytology in the setting of indeterminate biliary strictures is one of the most challenging dilemmas encountered in clinical practice. This scenario may lead to controversy in the management, delay in the diagnosis and treatment of malignancies and increased medical costs (9). The ability to differentiate benign from malignant etiologies is essential to prevent unnecessary interventions and surgeries. This study attempts to address this difficult issue and tries to further clarify the best approach to differentiate between patients with benign and malignant pathologies. In our study, we found that older age (≥65 years), presence of jaundice and underlying mass lesion on imaging are significant predictors of underlying malignancy in patients with biliary stricture and atypical cytology brush results.

In terms of patient-related factors, the univariable analysis showed that age ≥65 years, jaundice and weight loss were important predictors of malignancy. However, only older age and jaundice remained significant after adjusting for multiple potential confounders (adjusted OR 1.02 and 3.33, respectively). These results are concordant with previous studies that found age to be associated with pancreaticobiliary malignancy with the majority of affected patients in their sixth decade or older (15–17). In this study, patients with underlying malignancy were on average 11.4 years older compared to patients in the benign group (67.1 years versus 55.7 years, respectively). The degree of elevation of total bilirubin level is also an important predictor of malignancy. In patients with malignant biliary stricture etiologies, the average bilirubin level was almost 10-fold higher than the upper limit of normal. On the other hand, patients with benign etiologies had a bilirubin level less than five times the upper limit of normal. Hence, the bilirubin levels should be carefully assessed and incorporated into the decision-making process of patients with indeterminate biliary strictures.

Imaging criteria had even stronger predictive value when assessing for potential malignancy. In the unadjusted analysis, the presence of PD dilation, intrahepatic biliary dilation, double-duct sign and mass lesion were all significantly associated with underlying malignancy. However, adjusted analysis showed that only the finding of a mass lesion was strongly associated with underlying malignancy (adjusted OR 12.1). These findings highlight the importance of obtaining high-quality imaging in patients with indeterminate biliary strictures, since the finding of a mass lesion will increase the probabilities of diagnosing underlying malignancy. However, our results demonstrated that rare benign cases can also have mass lesions on radiology. Despite the advances in imaging modalities such as CT and MRI, these techniques have their own limitations and lower sensitivity especially for small lesions (<2 cm) (18). Even though EUS has an established role in the diagnosis of pancreaticobiliary malignancy and has been demonstrated to have significantly higher sensitivity and accuracy compared to CT and MRI in diagnosing pancreatic cancer (19), it remains an underutilized tool. In the current study, EUS was only used in 31.8% of the cases compared to 84.1% and 72.2% for CT and MRI, respectively. Given that >80% of lesions were either in the mid- or distal CBD where EUS is more accurate, EUS is likely to be very helpful in such scenarios. Therefore, incorporating EUS in the diagnostic algorithm of patients with indeterminate strictures has the potential to lessen the diagnostic uncertainty and improves the overall diagnostic yield. A proposed practical approach to patients with indeterminate biliary stricture is to perform EUS on all patients with mid- or distal CBD stricture and performing FNA of any mass lesion identified or at the site of the bile duct narrowing. The approach to proximal biliary strictures is more complicated given the limited sensitivity of EUS in such location. For such lesions, performing MRCP (if not already done) and/or referral to centers with access to cholangioscopy is the most appropriate next step.

PSC patients are one of the most challenging groups. These patients have a chronic inflammatory process affecting their biliary tree, which ultimately leads to biliary strictures. The chronic inflammatory process may also result in significant reactive cellular atypia that can be indistinguishable from neoplasia (20). Complicating things even further, patients with PSC are at significantly higher risk of developing biliary malignancies with an estimated 10 to 20% of patients eventually developing cholangiocarcinoma (21). Among the sub-group of patients with PSC in our cohort, 13.0% developed malignancy. Patients with PSC and malignancy were significantly older and had higher levels of serum Ca 19.9 compared to those with benign strictures. This confirms the importance of incorporating CA19-9 in the evaluation of PSC patients and suspicious biliary strictures. The optimal CA19-9 cutoff remains unclear, with some studies suggesting a level of 129 U/mL (22) and others 503 U/mL as the upper limit of normal for patients with PSC (23). Further studies are needed to clarify this specific issue.

Our study has a number of strengths. It is one of the largest studies to address this complicated and challenging clinical problem, increasing its statistical power. All of the included patients had long-term follow-up (minimum 6 months) to ensure the accuracy of our data. In addition, in our analysis, all the variables collected were adjusted for many confounders to further strengthen our findings and conclusions. All the cytology samples were examined by pathologists with years of experience in the interpretation of biliary cytology from a high-volume tertiary academic center, with a practice in which the majority of these cases would be reviewed within the group to limit the overuse of the ‘atypical’ diagnosis. Limitations to our study include the single center, retrospective design with all of its drawbacks and potential for bias. In particular, some of the clinical data was not available for all patients, which may affect the interpretation of some of our findings. On the other hand, the most relevant clinical and imaging variables were fully recorded, limiting the significance of the missing variables. Another limitation is the lack of ancillary testing (e.g., fluorescent in situ hybridization [FISH] testing) that may have helped to differentiate between benign and malignant etiologies. However, such tests are not widely available and suffer from their own limitations and hence are not available to most centers managing patients with biliary strictures (24). Finally, with the introduction of newer generation single-operator cholangioscopes that allow better visualization and targeted biopsy of suspicious biliary lesions, the diagnostic yield is improving (25). However, these devices are expensive, require experienced operator and are currently not available in many centers performing ERCP. Therefore, our study findings are more reflective of ‘real-world’ experience that can assist many physicians that may not have access to these devices.

An important area for improvement is better utilization of EUS in managing patients with indeterminate biliary strictures. One way to better coordinate EUS booking (if resources allow) is to have a nurse practitioner to follow the brush cytology results and book the patient for an EUS assessment if the results from the brush are ‘atypical’. This approach requires formal testing to assess the clinical- and cost-effectiveness of such strategy.

In conclusion, among patients with indeterminate biliary strictures and atypical biliary cytology results, older age, jaundice and the finding of mass lesion on imaging are significant predictors of underlying malignancy. These patients require careful and expedited assessment to rule out malignancy.

This manuscript has not been published previously in print or electronic format and is not under consideration by another publication or electronic medium.
